# Early Biliary Decompression Reduces Morbidity but Not Mortality in Acute Ascending Cholangitis

**DOI:** 10.7759/cureus.35989

**Published:** 2023-03-10

**Authors:** Emanuel A Shapera, Melissa Touadi, Steven Kaspick, Jennifer Choy-Shin, Mateusz Lapucha, Lauren Baumgarten, Matthew Johnson

**Affiliations:** 1 General Surgery, Olde Del Mar Surgical, San Diego, USA; 2 School of Medicine, University of South Florida, Tampa, USA; 3 Department of Surgery, University of San Francisco Fresno, Fresno, USA; 4 Department of Surgery, Kaiser Permanente, Lancaster, USA; 5 Department of Surgery, Wound Care Experts, Las Vegas, USA; 6 Department of Surgery, University of Tulane, Tulane, USA; 7 Department of Surgery, Fort Walton Beach Surgical Associates, Fort Walton Beach, USA

**Keywords:** cost benifit analysis, biliary decompression, morbidity and mortality, s: sepsis, endoscopy ercp, acute cholangitis

## Abstract

Background

Acute ascending cholangitis is a life-threatening infection due to biliary obstruction. Decompression via endoscopic retrograde cholangiography (ERC) or interventional radiologic (IR) drainage controls the source of the sepsis. Numerous studies have been published with conflicting data on whether earlier drainage affects morbidity and mortality. We sought to publish our experience at two Las Vegas community hospitals.

Methods

After IRB approval, over 4000 inpatient non-elective ERCs were analyzed between 2010 and 2019. Six-hundred and twenty-five patients met the 2018 Tokyo criteria for a “definitive diagnosis” of acute ascending cholangitis. A univariate and multivariate analysis was conducted to identify factors significantly associated with length of stay and mortality.

Results

On univariate analysis, patients who had drainage conducted within 24 hours had significantly shorter lengths of stay (p = 0.0012 95% CI [-88.1 to -21.8 hrs]), higher mean diastolic blood pressure (p=0.0029 95% CI [1.03 to 5.01 mm Hg]), and lower mean maximum temperature (p=0.0001 95% CI [-0.842 to -0.382 ^o^C]) when compared to patients who underwent decompression more than 24 hours after admission. There were no statistically significant differences in mortality between patients who underwent decompression within 24 hours of admission versus patients who underwent decompression beyond 24 hours of admission.

On multivariate analysis, earlier decompression reduced the length of stay for patients with mild (p<0.0001), moderate (p<0.0001), and severe cholangitis (p=0.0023). Mortality was significantly associated with the worsening severity of the cholangitis (moderate [p=0.0001] and severe [p<0.0001], but not mild disease) and the use of vasopressors.

Conclusions

Timely biliary decompression within 24 hours of admission significantly reduces the length of stay, pyrexia, and hemodynamic abnormalities. In addition, our data corroborate the 2018 Tokyo guidelines that correlate the severity of cholangitis with mortality.

## Introduction

Acute ascending cholangitis is a morbid condition afflicting patients secondary to calculous, malignant, or parasitical obstruction of the common bile or common hepatic duct, with gallstones being the most common cause in the United States [1]. Treatment is obtained by anatomical drainage either via endoscopic retrograde cholangiography (ERC) or interventional radiologic (IR) drainage.

The literature regarding the importance of urgent decompression (within 24 hours of admission) and non-urgent decompression (beyond 24 hours of admission) ERC drainage in reducing morbidity and mortality is conflicting. The largest study to date, utilizing a national inpatient sample of 107,253 patients with acute cholangitis, did not show a mortality benefit with urgent ERC but did show a reduction in length of stay (LOS) [2]. There was no subset analysis to account for disease severity, and their study was subject to the heterogeneity (institutional variation, laboratory measurements, etc.) that results when data from multiple hospital systems are utilized. A smaller institutional study of 177 patients, many with severe disease, demonstrated similar findings [3]. On the contrary, a similarly sized (166 patients) study by Ming Tan et al. from Odense University Hospital identified a statistically and clinically significant mortality benefit to urgent ERC (p=0.04; OR 0.24) [4]. To address the limitations in size and heterogeneity of these studies with their conflicting conclusions, we sought to publish our large dataset performed by the same group of endoscopists at two sister community hospitals.

## Materials and methods

Institutional Review Board approval was obtained. This was a retrospective study of a prospectively maintained database. Patients and the public were not involved in the design of this study, as it was considered routine to perform retrospective collection and univariate and multivariate analysis. Over 4000 inpatient non-elective decompressive procedures (ERC) were analyzed between 2010 and 2019 at two affiliated community hospitals in Las Vegas. Patients who met a “definitive diagnosis” of acute ascending cholangitis per the 2018 Tokyo Criteria [5] were included. Six hundred twenty-five patients were identified. Endoscopic reports were analyzed for each included patient to ensure the patient underwent endoscopic biliary decompression for acute ascending cholangitis. The etiology of the obstruction was stones and/or sludge. In some of these patients, a hepatobiliary malignancy generated an underlying partial obstruction that was superimposed by stones and sludge. All patients had at least 30 days of follow-up identified through their records. Pre-decompression clinical, laboratory, and demographic variables were tabulated. Times were recorded meticulously by the hour. Length of stay was calculated by determining the time interval (in hours) between the first and last recorded vital signs for a patient’s hospital course as recorded in nursing notes. The time to decompression from admission was calculated similarly by determining the time interval between the first recorded vital sign and the post-proceduralist’s (endoscopist’s) brief operative note, which is written immediately after the completion of any procedure. Patients who underwent biliary decompression within 24 hours of admission were categorized as having undergone “urgent decompression”, while patients who underwent decompression beyond 24 hours of admission were categorized as having undergone "non-urgent decompression".

Statistical analysis was conducted with MedCalc Statistical Software version 19.2.1 (MedCalc Software Ltd., Ostend, Belgium; https://www.medcalc.org; 2020). We conducted univariate analysis via independent samples t-test and chi-square test to determine differences in baseline and outcome variables for patients categorized as undergoing urgent and non-urgent biliary decompression. To account for confounding, we conducted multivariate analysis via stepwise logistic regression to determine which of these variables were significantly associated with mortality.

## Results

Patients who underwent urgent (≤24 hours) and non-urgent (>24 hours) decompression had similar baseline characteristics (Tables 1-2).

**Table 1:** Baseline Characteristics Before Decompression

Baseline Characteristics Before Decompression			
Patient characteristics	Urgent Decompression	Non-Urgent Decompression	P value
Total number	118	507	
Female	63 (52.9%)	278 (54.7%)	0.777
Age (years)	61.7	61.3	0.855
Presence of Charcot’s Triad	9	34	0.722
BMI (kg.m^-2^)	27.4	27.8	0.551
Diabetes	24	125	0.323
HTN	49	245	0.183
Pressor use	7	27	0.794
Mild cholangitis	54	258	0.317
Moderate cholangitis	53	189	0.125
Severe cholangitis	11	60	0.439

**Table 2:** Univariate Analysis of Admission Laboratory Work, Urgent vs. Non-Urgent Decompression AST: Aspartate transaminase, ALT: Alanine transaminase, ALP: Alkaline phosphatase, WBC: White blood cells

Univariate Analysis of Admission Laboratory Work, Urgent vs. Non-Urgent Decompression			
Pre-Decompression Labs, Mean	Urgent Decompression	Non-Urgent Decompression	P-value
WBC (µL^-1^)	14.3 ± 6.92	13.0 ± 6.31	0.064
Platelet Count (µL^-1^)	241 ± 83.3	223 ± 116	0.125
International Normalized Ratio	1.15 ± 0.40	1.26 ± 1.17	0.349
Sodium (mEq.L^-1^)	137 ± 3.98	137 ± 6.01	0.642
Creatinine (mg.dL^-1^)	0.939 ± 0.46	1.04 ± 0.91	0.245
AST (IU.L^-1^)	318 ± 731	290 ± 367	0.547
ALT (IU.L^-1^)	324 ± 601	297 ± 234	0.433
ALP (IU.L^-1^)	387 ± 306	377 ± 369	0.761
Albumin (g.dL^-1^)	3.90 ± 1.21	4.16 ± 8.60	0.740
Total Bilirubin (mg.dL^-1^)	5.20 ± 4.19	6.03 ± 6.09	0.159

On univariate analysis, patients who underwent urgent biliary decompression had significantly shorter LOS (129 vs. 184 hours, p = 0.0012, 95% CI [-88.1 to -21.8 hours]). Patients who underwent urgent biliary decompression had higher pre-decompression diastolic blood pressures (59.4 vs. 56.3 mmHg, p=0.0029 95% CI [1.03 to 5.01]), a lower pre-decompression maximum temperature (98.9 vs. 99.5 oC, p=0.0001 95% CI [-0.842 to -0.382]), a higher pre-decompression minimum temperature (97.4 vs. 97.1 oC, p=0.0001 95% CI [0.1613 to 0.4137]), and a lower pre-decompression heart rate (91 vs. 99 BPM, p=0.0001 95% CI [-11.9 to -3.88]) when compared to patients who underwent non-urgent biliary decompression (Table 3).

**Table 3:** Univariate Analysis of Outcome Variables

Univariate Analysis of Outcome Variables			
Outcome Variable	Urgent Decompression	Non-Urgent Decompression	P-value
Pre-Decompression Max Temp (^o^C)	98.9 ±0.88	99.5 ±1.20	0.0001, 95% CI (-0.842 to -0.382)
Pre-Decompression Min Temp (^o^C)	97.4 ±0.67	97.1 ±0.62	0.0001, 95% CI (0.1613 to 0.4137)
Pre-Decompression Systolic BP (mm Hg)	111 ±20.1	107 ±48.5	0.417, 95% CI (-12.6 to 5.25)
Pre-Decompression Diastolic BP (mm Hg)	59.4 ±10.5	56.3 ±9.78	0.0029, 95% CI (1.03 to 5.01)
Pre-Decompression Heart Rate (Beats per Minute)	91 ±20	99 ±20	0.0001, 95% CI (-11.9 to -3.88)
LOS (hours)	129 ±135	184 ±0.88	0.0012, 95% CI (-88.1 to -21.8)
Mortality	5 (4.24%)	36 (7.11%)	0.259
No. Discharged to Patient’s Home	101 (85.6%)	422 (83.4%)	0.561

There were 41 deaths, 19 among those with severe disease, 16 with moderate disease, and six with mild disease. Three by two chi-square test revealed a statistically significant difference in mortality between patients with severe (26.8%), moderate (6.6%), and mild cholangitis (1.9%) (p<0.0001) (Table 4). 

**Table 4:** Chi-Square 2-x3 Analyzing Mortality by Cholangitis Severity

Chi-Square 2-x3 Analyzing Mortality by Cholangitis Severity			
	Cases	Mortality	P-value
Mild Severity	312	6 (1.9%)	p < 0.0001
Moderate Severity	242	16 (6.6%)	
Severe Severity	71	19 (26.8%)	

On multivariate analysis, time to decompression was significantly associated with length of stay for patients with mild (p<0.0001), moderate (p<0.0001), and severe cholangitis (p=0.0023) (Table 5).

**Table 5:** Multivariate Stepwise Multiple Regression by Severity of Cholangitis for Length of Stay (Hours)

Multivariate Stepwise Multiple Regression by Severity of Cholangitis for Length of Stay (Hours)			
Variable Retained in Patients with:	Coefficient ± Standard Error	R partial	P-value
Mild Cholangitis			
Time to Decompression (hours)	1.23 ±0.140	0.452	<0.0001
Female Sex	-1.10 ±0.447	-0.141	0.0143
Moderate Cholangitis			
Time to Decompression (hours)	1.47 ±0.219	0.401	<0.0001
Sodium level (mEq.L^-1^)	-0.246 ±0.111	-0.143	0.0279
Alkaline Phosphatase (IU.L^-1^)	0.00223 ±0.001027	0.141	0.0306
Severe Cholangitis			
Time to Decompression (hours)	0.834 ±0.3343	0.296	0.0151
AST (IU.L^-1^)	0.0288 ±0.00625	0.497	<0.0001

Numerous laboratory and hemodynamic derangements were associated with increasing LOS (Table 6).

**Table 6:** Multivariate Stepwise Multiple Regression for Length of Stay (Hours), All Patients

Multivariate Stepwise Multiple Regression for Length of Stay (Hours), All Patients			
Variable Retained	Coefficient ±Standard Error	R partial	P-value
Age (Years Old)	0.0376 ±0.0138	0.114	0.0067
Creatinine (mg.dL^-1^)	1.05 ±0.309	0.141	0.0007
Diabetes	1.71 ±0.619	0.116	0.0059
Heart Rate (beats per minute)	0.0377 ±0.0146	0.108	0.0102
Pressor Use	5.49 ±1.14	0.186	<0.0001
Platelet Count (µL^-1^)	0.0115 ±0.00256	0.199	<0.0001
Pre-Decompression Max Temp (^o^C)	0.616 ±0.247	0.105	0.0127
Time to Decompression (hours)	1.08 ±0.129	0.333	<0.0001

Mortality was significantly associated with vasopressor use (p<0.0001), “moderate” severity disease (p<0.0001), and “severe” severity disease (p<0.0001). No statistically significant association was identified between mortality and timing of decompression for patients with “severe”, “moderate”, or “mild” severity acute cholangitis (Table 7). 

**Table 7:** Multivariate Stepwise Logistic Regression for all Patients for Mortality

Multivariate Stepwise Logistic Regression for all Patients for Mortality			
Variable Retained	Coefficient ±Standard Error	OR	P-value
Pressor Use	2.72 ±0.446	15.1 95% CI [6.31 – 36.2]	<0.0001
Severe Cholangitis	3.18 ±0.562	23.9 95% CI [7.95 – 72.1]	<0.0001
Moderate Cholangitis	1.46 ±0.556	4.30 95% CI [1.45 – 12.8]	0.0096

## Discussion

Length of stay is significantly reduced when biliary decompression is performed quicker; this finding is consistent with other studies [6,7]. This is likely due to the fact that persistent, uncontrolled sepsis from delayed biliary decompression results in multiorgan failure with an increase in the length of stay needed to treat it [8]. In this study, patients with non-urgent biliary decompression had a mean 55.5-hour greater LOS than patients who underwent urgent biliary decompression. This is greater than the time added by a mere delay in biliary decompression. This association held for cholangitis of any severity (mild, moderate, and severe). In an era of cost-conscious medicine, this paper will contribute to guiding patient care and resource allocation. Emergency access to staffing and equipment necessary to perform timely biliary decompression-resources that are often made scarce on weekends and evenings to save costs-should instead be made readily available. The expected reductions in patient morbidity and LOS should offset this cost. This paper therefore adds to the existing and growing consensus in the current literature advocating for urgent decompression and the resources necessary to maintain this capability at all times.

Other studies have produced contrary conclusions and should be addressed. Hakuta et al. studied 299 patients with acute ascending cholangitis and failed to demonstrate a benefit in LOS for patients with mild or moderate severity cholangitis who underwent timely biliary decompression. The authors conceded that their study chose an unusually short definition (< 12 hours) for “urgent” decompression as opposed to the 24 hours used by many other studies. Additionally, their study excluded patients with severe cholangitis; this group is the likeliest to benefit most from expedient biliary decompression, which would partly explain their failure to reject the null hypothesis.

In this study, patients decompressed beyond 24 hours were significantly associated with a faster heart rate, lower diastolic blood pressure, a higher maximum temperature, and a lower minimum temperature; both hypothermia and hyperthermia are markers of metabolic derangements associated with sepsis. As these vital signs are pre-decompression, this association can be attributed to an increase in septic morbidity. Patients awaiting septic source control likely developed further derangements in vital signs compared to patients who attained timely septic source control.

In regards to mortality, neither univariate nor multivariate analysis identified time to decompression as a statistically significant factor. This is congruent with the study of the large inpatient dataset by Malav P Parikh et al., where no mortality benefit was identified in 107,253 cases of acute ascending cholangitis for patients undergoing urgent versus non-urgent decompression, and with the study by Aboelsoud M et al. of 177 patients. Kiriyama et al. identified increasing mortality with the severity of cholangitis, congruent with the findings of this paper. In contrast to this paper, however, these authors did find a reduction in mortality among patients with timely biliary decompression-but only in those with moderate severity cholangitis, not mild nor severe severity [9]. Therefore, their study's conclusions did not differ much from the conclusions of this one.

In this study, there were 41 deaths among 625 cases, a mortality rate of 6.56%. This is congruent with Malav P Parikh et al., where mortality was < 5%. In contrast, Ming Tan et al. reported a mortality rate of 16.3%. Their inclusion criteria required an endoscopist to agree with a diagnosis of acute ascending cholangitis, introducing potential observer bias; it is possible their study included patients with more obvious and, therefore, severer cases of acute ascending cholangitis. This may explain their higher mortality rate and greater demonstrated benefit with expedient decompression, which patients with severe disease will likely benefit from the most. In contrast, Malav P Parikh et al. identified cases with ICD-9 codes, then ensured they fulfilled Tokyo criteria; there was no subjective inclusion based on endoscopist opinion. Unsurprisingly, our study attained similar mortality rates and conclusions regarding the effect of urgent decompression as the study by Malav P Parikh et al. Likely, there are many other factors impacting mortality, such as frailty, timely antibiotic administration, and fluid resuscitation, that are difficult to quantify and control via multivariate analysis. 

The identification of patients based on objective criteria is one of the strengths of this study, and its conclusions align with those of similarly designed retrospective studies. Although there are other retrospective studies, ours is the largest without the heterogeneity that national databases generate. By analyzing multiple variables, including lab work, vital signs, and severity of disease, our study has produced a thorough clinical analysis that few have been able to produce in many prior single-center or national database studies. Of note, we included patients who had underlying hepatobiliary malignancies contributing to biliary obstruction as long as their decompression was defined by the removal of stones and/or sludge and met the 2018 Tokyo criteria. Although the long-term prognosis of these patients differs markedly from that of patients with simple calculous biliary obstruction, their short-term survival, morbidity, and length of stay are largely inconsequential to the final outcome of their cancer, so these patients were not excluded. 

One question that remains unanswered by any study is how to define an appropriate timeframe to perform biliary decompression. The authors of this study analyzed differences between patients who underwent and did not undergo biliary decompression at 12 hours, 24 hours, and 48 hours and found similar conclusions: that a delay in decompression affected morbidity, not mortality. The authors of this study deferred publishing all these findings and chose 24 hours from admission as the point of difference because it would have tripled the already voluminous data available in this paper without changing its ultimate findings. The other major limitation of this study is the lack of randomization. However, it would be unethical to randomize patients to a treatment plan that involves a delay in septic source control; since it would put patients at risk of harm; this study aimed to define what that harm was, and the authors make their case that such harm has been defined, warranting timely biliary decompression as part of definitive septic source control to attenuate it. 

## Conclusions

Urgent decompression reduces septic morbidity in acute ascending cholangitis by reducing the length of stay and causing vital sign derangements. The severity of cholangitis and the use of vasopressors are both associated with mortality.
